# Fundamental social causes of inequalities in colorectal cancer mortality: A study of behavioral and medical mechanisms

**DOI:** 10.1016/j.heliyon.2020.e03484

**Published:** 2020-03-11

**Authors:** Sean A.P. Clouston, Julia Acker, Marcie S. Rubin, David H. Chae, Bruce G. Link

**Affiliations:** a Program in Public Health and Department of Family, Population, and Preventive Medicine, Stony Brook University, Stony Brook, NY, USA; b Family Community Medicine, University of California, San Francisco, CA, USA; c Section of Social and Behavioral Sciences, College of Dental Medicine, Columbia University, New York, NY, USA; d Center for Health Ecology and Equity Research, College of Human Sciences, Auburn University, Auburn, AL, USA; e Department of Sociology and School of Public Policy, University of California – Riverside, Riverside, CA, USA

**Keywords:** Demography, Epidemiology, Gastrointestinal system, Oncology, Physical activity, Public health, Social geography, Social inequality, Cancer epidemiology, Fundamental cause theory, Behavior, Medical sociology, Longitudinal

## Abstract

**Background:**

Fundamental cause theory posits that social conditions strongly influence the risk of health risks. This study identifies risk mechanisms that social conditions associated with socioeconomic status (SES) and race/ethnicity shape in the production of colorectal cancer (CRC) mortality.

**Methods:**

Two large datasets in the United States examining behavioral and medical preventive factors (*N* = 4.63-million people) were merged with population-level mortality data observing 761,100 CRC deaths among 3.31-billion person-years of observation to examine trends in CRC mortality from 1999-2012. Analyses examined the changing role of medical preventions and health behaviors in catalyzing SES and racial/ethnic inequalities in CRC mortality.

**Results:**

Lower SES as well as Black, Hispanic, Asian/Pacific Islander, and Native American race/ethnicity were associated with decreased access to age-appropriate screening and/or increased prevalence of behavioral risk factors. Analyses further revealed that SES and racial/ethnic inequalities were partially determined by differences in engagement in two preventive factors: use of colonoscopy, and participation in physical activity.

**Discussion:**

Social inequalities were not completely determined by behavioral risk factors. Nevertheless, a more equitable distribution of preventive medicines has the potential to reduce both the risk of, and social inequalities in, CRC mortality.

## 1 Introduction

Colorectal cancer (CRC) is the second leading cause of cancer-related deaths among American men and women, with an estimated 140,250 new diagnoses and 50,630 deaths from CRC in 2018 (Siegel et al., 2018). Over the last thirty years, age-adjusted CRC incidence and mortality decreased in the United States (U.S.) by over 45% and 50%, respectively (Welch and Robertson, 2016). These declines have been attributed to advancements in screening and early cancer detection, as well as to changes in behaviors (Edwards et al., 2010). Screening methods aim to identify and remove adenomatous (pre-cancerous) polyps and diagnose CRC at its earliest stages in order to allow timely initiation of treatment, while behavioral changes have made inroads into reducing incidence entirely. While it is promising that such innovations have prompted an overall decrease in CRC mortality, socioeconomic (Saldana-Ruiz et al., 2013) and racial/ethnic (Soneji et al., 2010) inequalities in CRC have emerged over the past 20 years, and appear to be growing.

Link and Phelan (1995) developed fundamental cause theory (FCT) to explain this conundrum, specifically suggesting that social inequalities in health arise in part because of human interventions. Specifically, the theory argues that SES broadly modifies access to flexible and fungible resources including knowledge, money, power, prestige, and beneficial social connections that can be deployed across different contexts to ensure better health outcomes for individuals and their social groups (Phelan et al., 2004; Phelan and Link, 2005). FCT states that SES predicts all-cause mortality because individuals, households, and larger communities can unequally access, obtain, and utilize resources in order to at once gain privileged access to protective factors and avoid risk factors. While it is clear that mechanisms linking social conditions to disease and mortality change over time (King and Bearman, 2011), there have been few studies that have clearly identified mechanisms for such changes.

An individual's risk of CRC mortality is determined, in part, by primary preventive efforts, including the avoidance of behavioral risk factors (e.g., physical inactivity), and secondary preventive efforts, including the use of blood stool testing and endoscopy (including for example colonoscopy, sigmoidoscopy, and proctoscopy) (Edwards et al., 2010). The *American Cancer Society* currently recommends that healthy eating, increased physical activity, and access to colorectal cancer screenings and appropriate diagnostic testing and treatments are all important to reducing the risk of CRC cancer (Kushi et al., 2012). Early CRC detection is accomplished through a combination of blood stool testing and colonoscopy. Blood stool testing detects approximately 13% of existing CRC types (Imperiale et al., 2004) and can help reduce CRC cancer mortality by approximately 16% when used appropriately (Hewitson et al., 2007). When used as a CRC screening tool, it generally requires follow-up testing, often utilizing colonoscopy (American Cancer Society, 2016). Colonoscopy is able to dramatically reduce the risk of incident colorectal cancer through the detection and removal of adenomatous polyps, which can give rise to CRC (Byers et al., 1997); a single sigmoidoscopy can reduce the risk of incident CRC by more than 50% (Atkin et al., 2010). U.S. guidelines for screening have, since 1980, focused on early detection efforts using endoscopy (Winawer et al., 1997), with current recommendations suggesting individuals aged 50 and older get screened for colorectal cancer using endoscopy with blood stool testing utilized afterwards for inter-colonoscopy monitoring purposes (American Cancer Society, 2016).

Behavioral change, which is often embodied through the modification or creation of healthful lifestyles (Cockerham, 2005), is a core component of the mechanisms linking social inequalities with CRC mortality. For example, higher education in childhood has been linked to lower rates of physical inactivity at midlife (Clouston et al., 2015), and low-SES parents are more likely to engage in obesogenic parenting practices that increase risk of obesity in childhood (Gibbs and Forste, 2014) and in young adulthood (Watts et al., 2016). In a seminal prospective cohort study, regular physical activity was associated with a 50% reduction in the risk of colon cancer while obesity was associated with a three-fold increase in that risk (Giovannucci et al., 1995). More recent results continue to suggest a core role for physical inactivity revealing, for example, that being sedentary post-diagnosis results in a 30% reduction in CRC survival (Campbell et al., 2013) and that post-diagnostic physical activity improves survival independently of pre-diagnostic levels of activity (Arem et al., 2015).

Critical to understanding the nature of social inequalities in CRC is whether SES is both mediated by these mechanisms, and whether it moderates their effectiveness. Previous studies have found that social inequalities are not evident in CRC mortality when diagnoses are received at more advanced stages (American Cancer Society, 2013), or in the type or intensity of treatment once patients are receiving clinical care (Frederiksen et al., 2009). Instead, as detailed below, research has found that social inequalities are evident in the uptake of CRC screening and in behaviors implicated in CRC risk such as physical inactivity suggesting that inequalities may arise from both behavioral and medical efforts to improve health and reduce the risk of CRC. However, while behavioral risk factors are both monotonic in direction and independent, medical screening factors are not. Behavioral risk factors are monotonic (or unidirectional) in nature because they specify that individuals should seek to improve health by engaging in more/less of a preventive/risky behavior. For example, the cancer guidelines suggested more exercise accompanied by less obesity rather than suggesting an optimal weight or a particular exercise routine. They are independent because the avoidance of each is believed to operate irrespective of the others: exercising, while linked to smoking and obesity, reduces CRC risk irrespective of other behaviors even of changes in body mass (Thune and Lund, 1996).

Medical screening factors, though often categorized as similar in nature and in result, have qualitatively different outcomes in terms of their ability to reduce CRC mortality risk. While both blood stool testing and colonoscopy are categorized generically as “screening techniques,” blood stool testing *can only aid in the diagnosis of existing CRC* while a single colonoscopy can both diagnose more existing CRC and can also uniquely prevent, through precancerous polyp removal, a large proportion of incident CRCs from forming for as much as ten years (Atkin et al., 2010). However, SES inequalities have been extensively linked to numerous barriers in CRC screening including, for example, fear of pain or cancer diagnosis (Jones et al., 2010), and fears about “tempting fate” and about the potential for increased anxiety about CRC after screening (Smith et al., 2016), reluctance on the part of providers in providing recommendations (Weiner et al., 2017), and lack of access to, knowledge about, and provider recommendations for preventive medicine are well documented obstacles to colonoscopy uptake (Green et al., 2008). Social inequalities can therefore emerge when blood stool testing is used alone or is used first because it allows time to elapse between screening and preventive efforts, and if used alone it can lead those who screen negative to feel safe thereby delaying further screening. This can be dangerous; in one study of such a screening program, only half of those who screened positive using fecal blood testing alone ever received follow-up testing or treatment, even when such follow-up was available to patients free of charge (Fisher et al., 2006). Thus, when individuals might reasonably understand that CRC screening is important, they might either select or be steered towards less effective screening techniques (blood stool testing) for convenience or due to costs (Roy et al., 2006), resulting in delayed access to effective technologies when the most effective method is a combination of colonoscopy followed by regular blood stool testing and follow-up colonoscopies every 5–10 years to monitor for previously unidentified cancers or developing polyps (Bibbins-Domingo et al., 2016).

No study to date has examined the role of common health behaviors in mediating the SES to CRC mortality relationship. We propose that greater social advantage will predict the likelihood of utilizing screening technologies, and will also increase the *quality* of screening, and thus the likelihood that, and the rate at which, such technologies might beneficially reduce risk of CRC mortality. Relative to those occupying more advantaged positions, people in less advantageous positions are not only likely to lack access to new information about preventions, but also to be less informed about the correct use of such preventive measures when they are available.

## 2 Method

### 2.1 Analytic strategy

The two data sets we use are 1) annual mortality counts collated by race/ethnicity, age, sex, and county of residence derived from the Compressed Mortality File (CMF) and 2) behavioral risk factors and preventive usage drawn from the Behavioral and Risk Factor Surveillance Study (BRFSS). Because the risk of CRC is extremely low for individuals under age 25, we excluded those under age 25. Since the race/ethnicity codes were comparable starting in 1999, we limited analyses to the years 1999–2012. We independently examined the role of SES in determining behavioral and preventive usage and then merged area-level usage rates with CRC mortality rates in order to examine whether inequalities were greater in areas and periods where healthy behaviors and preventions were more common.

### 2.2 County-level mortality data

To understand the risk of mortality, we utilized the CMF (https://wonder.cdc.gov), a publicly-available dataset available from the National Center for Health Statistics (2012) that retrieves cause of death, age, race, and county of residence of all U.S. residents. To identify CRC deaths for the years 1999–2012, we used International Classification of Disease codes (Version 10: C18–C21). Data incorporated 54.4 thousand CRC deaths per year from a total of 3.31 billion person-years of observation.

### 2.3 Individual-level prevention data

Since 1984, the Behavioral Risk Factors Surveillance Study (BRFSS; www.cdc.gov/brfss) has annually surveyed cross-sectional samples of the U.S. population to assess and describe known behavioral and medical risk factors for a broad range of diseases (Centers for Disease Control and Prevention (CDC), 2013). In 1998, the BRFSS began measuring use of colonoscopy (or sigmoidoscopy/proctoscopy). We excluded those living in regions (including Puerto Rico) that were not available in the mortality database, and because there are no missing data on demographics in the CMF database, we also excluded BRFSS respondents with missing values on race, sex, income, or education. From that, we removed those who did not have complete data on sociodemographics or smoking status, leaving a final analytic sample of 4,633,564 person-years.

### 2.4 Data merging

State of residence is critical to this analysis because individuals seek screening opportunities within their own state and are ultimately influenced by state-level efforts to organize, advertise, and provide screening. Since states are the ultimate geographic unit in which healthcare policies are made and in which healthcare resources are organized, analysis of mechanisms was completed using state-level estimates, which were then merged onto mortality data. Data were merged using race-, year-, and state-specific usage rates (%ages) for each prevention type merged to the underlying mortality data at the state-level. Because race was never reported as unknown in the mortality data, merged data included only reported race/ethnicity. Merged data were analyzed to examine whether behavioral and medical preventive efforts were effective in both explaining and exacerbating socioeconomic inequalities in CRC mortality. Because data are available from BRFSS for 11 years prior to data being available on the full range of races/ethnicities currently used in the research, we completed full analyses first using the full range of years (1988–2012) and then again using the more limited year-range (1999–2012). Differences in results were minimal so only results for analyses of 1999–2012 were presented.

### 2.5 Measures

#### 2.5.1 Preventive factors

There are known behavioral risk factors and available medical interventions that influence the risk of CRC. Behavioral risk factors include smoking, obesity, and sedentary behaviors. Medical preventions for colorectal cancer include are, as noted above, colonoscopy and blood stool testing. In the BRFSS, individuals were asked whether they currently smoke cigarettes. Obesity rates were derived from self-reported height (in centimeters) and weight (in kilograms). Body mass index (BMI) was calculated as kg/m^2^; individuals with a BMI of 30 or more were categorized as obese. Physical activity was measured by asking whether respondents had, in the past month, participated in any leisure-time physical activities, such as running, bicycling, gardening, or brisk walking. Diet was assessed using a six-item measure of fruit and vegetable intake that determined the frequency of fruit juice, fruit, beans/legumes, dark green vegetables, orange vegetables, and other vegetable consumption over the past month (Control and Prevention, 2015). Finally, respondents were asked whether they had ever had 1) a colonoscopy or 2) a blood stool test.

#### 2.5.2 Socioeconomic status

SES was measured at the county-level using five indicators (% of households living below the poverty line, % with more than 12 years of education, % with fewer than 9 years of education, % white collar occupations, and % owning a telephone) available from the decennial census. Principal components analysis was used to create a scale (Cronbach's α = 0.92). Intercensal years were linearly interpolated.

Education and Household Income were measured at the individual level in the *BRFSS.* Specifically, *education* identifies whether individuals had fewer than 9 years, 9–11 years, 12 years or equivalent, 1–3 years of college or technical schooling, or a 4-year college degree or more. Individuals were further asked whether their household annual income was categorized into households earning <$50,000, $50–74,999, or ≥$75,000.

Race/Ethnicity was categorized using standard census categories: White, African American, Asian/Pacific Islander, or American Indian/Alaskan Native. In the BRFSS alone, individuals also said Other, or Don't know/refused. Individuals were also asked to report Hispanic descent. However, most individuals of Hispanic descent (78.3%) responded that they did not know their own race. Seeing that as a signal that Hispanic descent is treated similarly to race in the minds of many Hispanics, we created a seventh racial/ethnic category: “Hispanic.”

Year of death and sex of decedent were recorded on death certificates and available in the CMF data. Year and month of interview is noted in the BRFSS file. In both cases, year was centered (Year-2000) to facilitate interpretation. Age, sex, and race were available at the individual level for BRFSS and CMF data files. In the CMF file, age was aggregated into ten-year age groups and thus analyses of BRFSS data utilized the same approach.

## 3 Statistical analysis

### 3.1 Descriptive analyses

Descriptive analyses provided average prevalence rates for risk and protective factors by year weighted to the U.S. population aged 25 and older. Numbers of CRC deaths per year were provided alongside midyear population estimates derived from the decennial census. Crude mortality rates were estimated by dividing the number of deaths by the total population and multiplying by 100,000.

### 3.2 Modeling risk and protective factor prevalence

We used logistic regression to estimate associations between SES and five identified prevention methods and risk factors for CRC. Specifically, we examined the log-odds that respondents answered yes to the use of medical preventions as specified in Eq. (1) below:

(1) $$\ln\left(\frac{\pi }{1-\pi }\right)=\beta_{1}E_{irt}+\beta_{2}I+\beta_{3}t+\left(\beta_{4}E_{irt}+\beta_{5}I\right)t+\beta_{k}X_{k}$$

where adjustments for education (E), income (I), time (*t*) and an array of demographic indicators (***X***_***k***_) were incorporated. In the above equation, we also examined how socioeconomic factors might moderate changes over time.

To examine differences in patterns of access to screening methods we replicated the model shown above using multinomial logistic regression (Long and Freese, 2006). Multinomial logistic regression allows researchers to examine overlapping predictors of multiple mutually exclusive but unordered categories in relation to a single reference category (here not having screening). Odds ratios and 95% confidence intervals were reported; adjusted Pseudo-R^2^ were used to indicate model fit (McFadden, 1973).

### 3.3 Data merging

To merge BRFSS estimates with CMF data, we used multivariable logistic regression to estimate the state-level likelihood of each prevention or risk factor weighted to the U.S. population using weights that accounted for yearly changes in sample size. We then used those models to estimate the marginal prevalence of prevention and risk factors for each state by race, sex, and 10-year age group.

### 3.4 Modeling mortality

To model CRC mortality rates, we used negative binomial regression, relying on midyear population counts to model the population at risk of death. Negative binomial regression is used to model count data that follow a Poisson process. Unlike Poisson, negative binomial regression is robust to over-dispersion, which is common when modeling mortality data (Gardner et al., 1995). The expected value of Y (here mortality counts) is calculated using the following equation, where δ is the measure of unobserved heterogeneity $\mathrm{Pr}\left(Y=y|X=x,\;\text{Δ}=\delta \right)=\frac{\text{Γ}\left(\lambda +y\right)}{\text{Γ}\left(\lambda \right)\text{Γ}\left(y+1\right)}{\left(\frac{\delta }{1+\delta }\right)}^{y}{\left(1+\delta \right)}^{-\lambda }$. In practice, negative binomial models estimate ln(λ)=βX**,** where the expected value of Y can be derived as E(y)=δλ and Var(y)=(1+δ)δλ. Since we were interested in modeling changes in the associations between CRC mortality and both SES and Race over time, we used Eq. (2) below:

(2) $$\text{Ln}\left(\lambda \right)=\beta_{1}SES+\beta_{2}t+\beta_{3}S_{s}+\beta_{4}X_{s}+\beta_{5}O_{s}+\beta_{6}C_{s}+\beta_{7}B_{s}+\beta_{8}SES*\left(S_{s}+X_{s}+O_{s}+C_{s}+B_{s}+t\right)+\beta_{11}a+\beta_{12}g+\beta_{13}R+\beta_{14}Y+\gamma_{0c}$$

where smoking (S), exercise (X), obesity (O), colonoscopy (C), and blood stool testing (B) are measured at the state-level (using BRFSS data), noted by the subscripted “s.” Year (Y) was incorporated to capture unobserved temporal reductions in CRC mortality. To capture unobserved contextual differences at the state level, we utilized state-specific (*γ*_*0C*_ above) random intercepts (Diez-Roux, 2000). Mortality rate ratios (MRRs), 95% confidence intervals, and *p*-values were provided. When data are not over-dispersed (*α* = 0), negative binomial regression reduces to Poisson regression; *α* was estimated and likelihood ratio tests were used to examine whether data are over-dispersed (*α*≠0). Models adjusted for age (a) and sex (g). Midyear population counts were used to model exposure and analyses were limited to counties with non-zero populations. Model fit was assessed using the Pseudo-*R*^*2*^; R^2^-change was used to compare nested models. Accompanying *p*-values were derived from *F*-tests.

## 4 Results

### 4.1 Descriptive results [Table 1, Figure 1]

Crude CRC mortality rates declined over time from a high of 12.6/100,000 person-years in 1988 to a low of 8.6/100,000 person-years in 2010 (Supplemental Table S1). While rates declined, numbers of CRC deaths were relatively stable over time (a pattern due predominantly to increases in population size). CRC mortality rates were higher among Whites than African Americans in 1999 (risk difference = -1.72/100,000), but this difference reduced in size by half by 2012 (risk difference = -0.82/100,000).

Data from the BRFSS revealed a sample that (Table 1), after being weighted to the U.S. population, was well educated, majority White, and at midlife during observation. These data also revealed that only approximately half of respondents had received a colonoscopy while only 40% had received a blood stool test. Additionally, more than one-quarter of respondents were obese, and more than three-quarters reported never exercising.

**Table 1:** Sample characteristics from the Behavioral Risk Factors Surveillance Study 1999–2012.

Variable Name	Category	Mean	SD
Age in years		48.70	15.47
		%	
Male sex		49.40	
Education	Primary School	4.32	
	Secondary School	6.95	
	High School Degree	54.59	
	University Degree	34.14	
Income	Less than $35,000	38.94	
	$35,000–50,000	16.2	
	More than $50,000	44.86	
Race/Ethnicity	White	71.05	
	African American	9.82	
	Hispanic	12.36	
	Asian/Pacific Islander	3.2	
	First Nations	1.01	
	Other	0.68	
	Don't Know/Refused	1.88	
Health Behaviors	Received Colonoscopy	54.60	
	Received Stool Testing	40.22	
	Smoker	20.19	
	Obese	26.50	
	Never Exercises	75.08	
	Low Fruit Intake	38.89	

**Figure 1:**
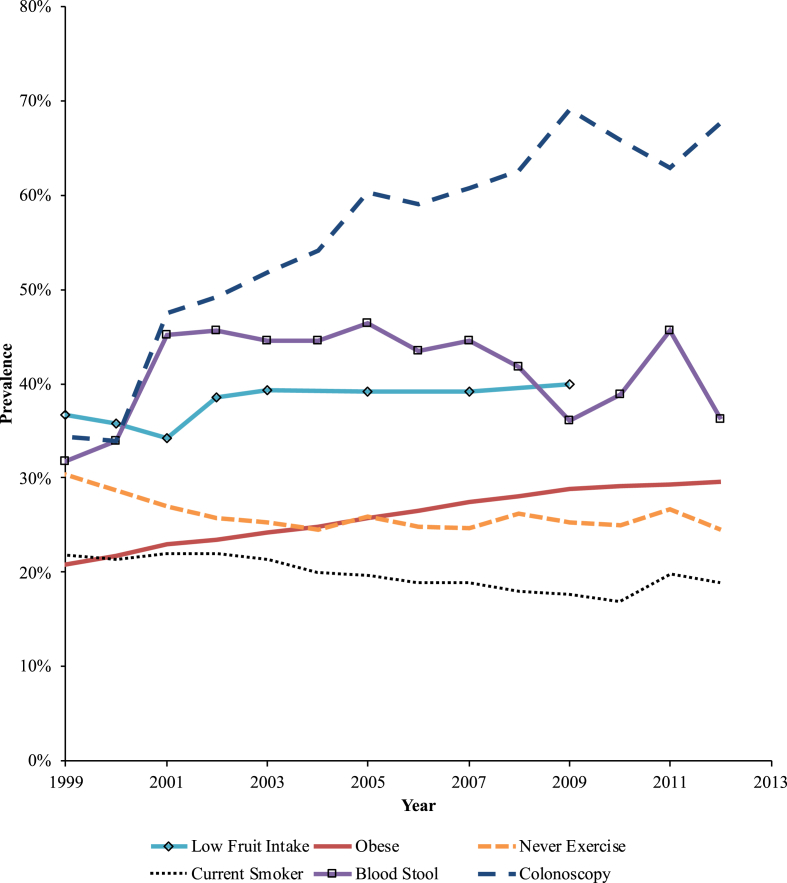
Prevalence of behaviors linked to colorectal cancer risk and use of screening techniques weighted to the United States resident population aged 25 and older, Behavioral and Risk Factor Surveillance Study 1999–2012.

Examining yearly trends in prevention uptake (Figure 1) revealed that use of CRC preventive methods improved over time. There was a decrease in sedentary behavior and smoking (Figure 1) and an increase in colonoscopy use.

### 4.2 Individual-level SES and preventive behaviors [Table 2]

Associations between education, income, and race/ethnicity with measures of prevention (Table 2) revealed that education and income, both domains of SES, were consistently associated with higher access to preventive technologies and lower risk of smoking, physical inactivity, and obesity, and low fruit/vegetable intake. Racial/ethnic disparities were also evident. For example, while Black race was not associated with prevention usage, it was predictive of having lower smoking rates, more physical inactivity, higher obesity, and lower fruit intake. In contrast, Asian/Pacific Islanders were less likely to receive colonoscopy or blood stool testing, but were also less likely to be smokers, to be physically inactive, obese, and to have low fruit/vegetable intake suggesting that race/ethnicity is not unidirectional in predicting health disparities. Longitudinal analyses showed that socioeconomic gradients grew over time for colonoscopy usage. Changes in racial/ethnic inequalities also showed consistent reductions such that Blacks, for example, appeared to improve relative to Whites across behavioral (smoking, physical inactivity, obesity, and low fruit intake) and preventive medical (colonoscopy and blood stool testing) factors.

**Table 2:** Odds ratios and 95% confidence intervals derived from logistic regression analyses examining individual-level associations between preventions and educational attainment and income both at baseline and over time, Behavioral Risk Factors Surveillance Study 1988–2012.

	Model 1: Colonoscopy		Model 2: Blood Stool Test		Model 3: Current Smoker		Model 4: Never Exercise		Model 5: Obese		Model 6: Low Fruit Intake	
	OR	95% CI	OR	95% CI	OR	95% CI	OR	95% CI	OR	95% CI	OR	95% CI
**Intercepts**												

**Race/Ethnicity**												
White	1.000		1.000		1.000		1.000		1.000		1.000	
Black	1.002	0.943, 1.065	0.901	0.848, 0.958	0.721	0.695, 0.747	1.325	1.279, 1.372	1.704	1.651, 1.759	1.136	1.084, 1.191
Hispanic	0.884	0.811, 0.964	0.494	0.451, 0.541	0.509	0.485, 0.535	1.710	1.636, 1.786	1.179	1.13, 1.23	0.940	0.887, 0.997
API	0.583	0.49, 0.694	0.288	0.238, 0.349	0.590	0.53, 0.657	1.987	1.809, 2.183	0.312	0.275, 0.353	0.908	0.81, 1.017
AIAN	1.040	0.879, 1.231	0.797	0.664, 0.955	1.455	1.328, 1.594	1.205	1.093, 1.328	1.343	1.225, 1.473	0.926	0.819, 1.047
Other	0.768	0.629, 0.939	0.672	0.543, 0.831	1.222	1.074, 1.39	1.284	1.123, 1.469	0.935	0.815, 1.073	1.055	0.887, 1.255
Don't Know/Refused	1.038	0.916, 1.176	0.796	0.706, 0.897	1.218	1.123, 1.321	1.199	1.104, 1.302	1.163	1.078, 1.255	0.936	0.834, 1.05
**Education**												
Primary School	1.000		1.000		1.000		1.000		1.000		1.000	
Secondary School	1.155	1.05, 1.27	1.300	1.187, 1.423	1.634	1.519, 1.758	0.826	0.769, 0.886	0.953	0.888, 1.022	0.912	0.825, 1.008
High School Degree	1.396	1.288, 1.514	1.850	1.713, 1.999	0.968	0.905, 1.035	0.504	0.474, 0.536	0.803	0.754, 0.854	0.740	0.678, 0.807
University Degree	1.829	1.679, 1.992	2.244	2.067, 2.436	0.431	0.402, 0.463	0.275	0.258, 0.294	0.564	0.528, 0.602	0.476	0.435, 0.521
**Income**												
Less than $35,000	1.000		1.000		1.000		1.000		1.000		1.000	
$35,000–50,000	1.213	1.161, 1.268	1.218	1.167, 1.27	0.755	0.734, 0.776	0.691	0.671, 0.712	0.887	0.863, 0.912	0.913	0.88, 0.948
More than $50,000	1.524	1.465, 1.585	1.541	1.484, 1.601	0.539	0.525, 0.553	0.486	0.473, 0.5	0.758	0.74, 0.777	0.825	0.799, 0.853
**Slopes**												

**Race/Ethnicity**												
White	1.000		1.000		1.000		1.000		1.000		1.000	
Black	1.005	0.998, 1.012	1.010	1.003, 1.017	1.001	0.996, 1.005	0.989	0.985, 0.993	0.994	0.99, 0.998	0.988	0.98, 0.996
Hispanic	0.986	0.976, 0.996	1.027	1.016, 1.038	0.975	0.969, 0.981	0.966	0.961, 0.971	0.996	0.991, 1.001	0.994	0.985, 1.004
API	1.005	0.986, 1.025	1.089	1.065, 1.113	0.994	0.981, 1.008	0.970	0.959, 0.981	1.011	0.997, 1.026	0.971	0.954, 0.989
AIAN	0.972	0.953, 0.991	1.007	0.986, 1.028	0.991	0.979, 1.002	0.996	0.984, 1.008	1.000	0.989, 1.012	0.990	0.971, 1.01
Other	0.999	0.973, 1.025	1.017	0.99, 1.045	0.969	0.951, 0.987	0.986	0.968, 1.004	0.996	0.978, 1.014	0.945	0.916, 0.975
Don't Know/Refused	0.984	0.969, 0.998	1.018	1.004, 1.032	0.994	0.984, 1.004	0.990	1, 0.949	0.999	0.99, 1.008	0.986	0.969, 1.003
**Education**												
Primary School	1.000		1.000		1.000		1.000		1.000		1.000	
Secondary School	1.008	0.996, 1.02	1.005	0.993, 1.017	1.009	0.999, 1.018	1.014	1.006, 1.023	1.006	0.998, 1.015	1.025	1.008, 1.043
High School Degree	1.020	1.009, 1.03	0.997	0.987, 1.008	1.005	0.996, 1.014	1.024	1.016, 1.031	1.018	1.011, 1.026	1.018	1.003, 1.033
University Degree	1.022	1.011, 1.034	0.995	0.985, 1.006	0.988	0.979, 0.997	1.025	1.017, 1.033	1.013	1.005, 1.021	1.016	1.001, 1.032
**Income**												
Less than $35,000	1.000		1.000		1.000		1.000		1.000		1.000	
$35,000–50,000	1.015	1.01, 1.021	0.995	0.99, 1	0.987	0.984, 0.991	1.004	1, 1.008	1.003	0.999, 1.007	0.990	0.984, 0.996
More than $50,000	1.019	1.015, 1.024	0.978	0.973, 0.982	0.973	0.97, 0.977	1.003	0.999, 1.006	1.005	1.002, 1.008	0.983	0.978, 0.989
Pseudo R^2^	0.109		0.068		0.095		0.035		0.042		0.042	
Sample Size	1262712		1262966		3789794		3678035		3690211		1537275	

**Note:** Models also adjust for pre/post MSTF, year, pre/post MSTF x year, age, age^2^, sex, and integrate state-level random intercepts. AIAN: American Indian/Alaskan Native; API: Asian/Pacific Islander census categories.

### 4.3 Differences in quality

Next, we considered the potential differences in type of treatment used (Table 3) using multinomial logistic regression to compare predictors of having either only a blood stool test (Outcome 1), only a colonoscopy (Outcome 2), or both a blood stool test and colonoscopy screening (Outcome 3); models are further broken into time-invariant analyses of intercepts, and time-variant analyses of slopes. Time-invariant analyses in outcome 1 (marked as “intercepts”) showed a small gradient such that those with higher education and above median income were more likely to use blood stool testing without colonoscopy. There was a net advantage for Blacks when accessing both blood stool and colonoscopy testing alone, as well as a net disadvantage for Hispanics, Pacific Islanders, and individuals of “other” race. Gradients were stronger when examining access to both screening methods, with both education and income predicting larger increases in access and with each level of education and income showing significant overall benefits.

**Table 3:** Odds ratios and 95% confidence intervals derived from multinomial logistic regression analyses examining individual-level associations between preventions and educational attainment and income both at baseline and over time, Behavioral Risk Factors Surveillance Study 1988–2012.

	Outcome 1: Blood Stool Testing without Colonoscopy		Outcome 2: Colonoscopy without Blood Stool Testing		Outcome 3: Both Blood Stool and Colonoscopy	
	OR	95% CI	OR	95% CI	OR	95% CI
**Intercepts**						

**Race/Ethnicity**						
White	1.000		1.000		1.000	
Black	1.100	(1.042–1.161)	1.064	(1.013–1.117)	1.037	(0.973–1.106)
Hispanic	0.760	(0.709–0.814)	0.979	(0.917–1.044)	0.665	(0.608–0.727)
Pacific Islander	0.569	(0.496–0.653)	0.687	(0.601–0.786)	0.327	(0.27–0.396)
First Nations	0.927	(0.776–1.107)	0.984	(0.846–1.144)	0.838	(0.687–1.023)
Other	0.797	(0.647–0.982)	0.804	(0.669–0.967)	0.646	(0.504–0.828)
Don't Know/Refused	0.923	(0.785–1.085)	1.094	(0.948–1.263)	0.806	(0.697–0.931)
**Education**						
Primary School	1.000		1.000		1.000	
Secondary School	1.189	(1.094–1.292)	1.019	(0.947–1.096)	1.356	(1.239–1.484)
High School Degree	1.429	(1.331–1.535)	1.191	(1.12–1.267)	1.957	(1.814–2.111)
University Degree	1.646	(1.525–1.777)	1.538	(1.439–1.643)	2.822	(2.605–3.059)
**Income**						
Less than $35,000	1.000		1.000		1.000	
$35,000–50,000	1.064	(1.024–1.106)	1.126	(1.087–1.167)	1.339	(1.283–1.399)
More than $50,000	1.245	(1.198–1.293)	1.541	(1.49–1.594)	1.823	(1.751–1.898)
**Slopes**						

**Race/Ethnicity**						
White	1.000		1.000		1.000	
Black	0.984	(0.976–0.992)	0.998	(0.992–1.004)	0.991	(0.983–1)
Hispanic	0.963	(0.953–0.974)	0.997	(0.989–1.005)	0.967	(0.955–0.978)
Pacific Islander	0.977	(0.956–0.998)	1.008	(0.993–1.024)	1.028	(1.005–1.052)
First Nations	0.985	(0.958–1.013)	0.979	(0.961–0.998)	0.981	(0.957–1.006)
Other	0.993	(0.958–1.029)	0.995	(0.969–1.021)	0.994	(0.959–1.03)
Don't Know/Refused	1.011	(0.991–1.032)	0.983	(0.966–0.999)	1.004	(0.988–1.021)
**Education**						
Primary School	1.000		1.000		1.000	
Secondary School	1.013	(0.998–1.028)	1.013	(1.002–1.023)	1.018	(1.004–1.032)
High School Degree	1.035	(1.022–1.049)	1.028	(1.019–1.036)	1.031	(1.019–1.043)
University Degree	1.034	(1.019–1.048)	1.030	(1.021–1.04)	1.033	(1.021–1.046)
**Income**						
Less than $35,000	1.000		1.000		1.000	
$35,000–50,000	1.013	(1.007–1.019)	1.023	(1.018–1.028)	1.011	(1.005–1.018)
More than $50,000	0.998	(0.992–1.004)	1.014	(1.01–1.019)	1.002	(0.997–1.007)
Sample size	1,390,666					
Pseudo-R^2^	0.091					

**Note:** Models also adjust for pre/post MSTF, year, pre/post MSTF x year, age, age^2^, sex, and integrate state-level random intercepts.

Time-variant slope analyses (marked as “slopes” in Table 3) suggested that higher education was associated with increases in access to colonoscopy over time. Furthermore, both educational and income gradients in colonoscopy and blood stool testing increased over time. Racial disparities were also more pronounced with regard to having both screening measures, with drastic differences emerging for Asian/Pacific Islanders and for Hispanic individuals.

### 4.4 State-level SES, prevention, and CRC mortality [Table 4]

To understand how such inequalities might influence CRC mortality, we examined associations between social inequality and CRC mortality risk (Table 4). We began by examining models of change in SES and Racial/ethnic disparities over time (Model 1). Results revealed that higher SES was associated with lower risk of CRC mortality, and also suggested that when adjusting for geographic location, age, and sex, Blacks, Hispanics, and Asian/Pacific Islanders were at lower risk, while Native Americans were at higher risk, of CRC mortality. Longitudinal analyses suggested that individuals of Hispanic ethnicity became increasingly disparate from Whites when controlling for SES, while higher SES was associated with an increasing disparity in CRC mortality over time.

**Table 4:** Mortality rate ratios and 95% confidence intervals examining area-level risk of colorectal cancer mortality by socioeconomic status and Race/Ethnicity, and examining the role of preventive efforts, Compressed Mortality File 1999–2012.

	Model 1	Model 2	Model 3
	MRR (95% CI)	MRR (95% CI)	MRR (95% CI)
**Socioeconomic Status**	0.971 (0.963, 0.979)	0.968 (0.96, 0.976)	0.952 (0.942, 0.962)
**Race/Ethnicity**			
White	1.000	1.000	1.000
Black Race	0.875 (0.645, 1.187)	1.197 (0.864, 1.658)	1.332 (0.964, 1.84)
Hispanic	0.626 (0.598, 0.656)	1.478 (1.269, 1.72)	1.573 (1.352, 1.829)
Asian/Pacific Islander	0.236 (0.132, 0.422)	0.361 (0.19, 0.683)	0.346 (0.183, 0.654)
Native American	2.342 (1.422, 3.858)	4.498 (2.412, 8.387)	4.846 (2.591, 9.061)
**Male**	1.407 (1.394, 1.421)	1.28 (1.15, 1.426)	1.345 (1.231, 1.47)
**Year**	0.967 (0.962, 0.973)	0.963 (0.955, 0.971)	0.963 (0.955, 0.971)
**Socioeconomic Status x Year**	0.994 (0.993, 0.995)	0.994 (0.993, 0.995)	0.997 (0.995, 0.999)
**Race/Ethnicity x Year**			
White	1.000	1.000	1.000
Black Race	0.986 (0.943, 1.031)	0.977 (0.933, 1.023)	0.977 (0.934, 1.023)
Hispanic	1.019 (1.013, 1.026)	1.008 (1.002, 1.014)	1.008 (1.002, 1.014)
Asian/Pacific Islander	1.049 (0.977, 1.127)	1.065 (0.985, 1.151)	1.061 (0.983, 1.147)
Native American	0.959 (0.895, 1.027)	0.932 (0.858, 1.013)	0.934 (0.858, 1.016)
**Mechanisms**			
Colonoscopy		0.662 (0.631, 0.696)	0.669 (0.637, 0.702)
Blood stool test		1.316 (1.282, 1.35)	1.23 (1.199, 1.262)
Smoking		0.761 (0.734, 0.789)	0.733 (0.707, 0.76)
Never Exercise		0.519 (0.479, 0.562)	0.502 (0.464, 0.544)
Obesity		1.236 (1.152, 1.326)	1.185 (1.105, 1.271)
Low Fruit Intake		1.038 (0.923, 1.167)	1.04 (0.926, 1.168)
**Socioeconomic Mechanisms**			
Colonoscopy			0.977 (0.96, 0.996)
Blood stool test			1.157 (1.136, 1.178)

**Note:** Models also adjust for age in years and sex and integrate state-level random intercepts.

Time (in years) was associated with expected reductions in CRC mortality. Results examining the role of preventions (Model 2) revealed associations between colonoscopy uptake and physical activity with lower risk of CRC mortality. However, blood stool testing and obesity were associated with higher risk of CRC mortality. Notably, associations between Hispanic ethnicity and CRC mortality inverted upon adjustment for risk factors, such that Hispanics were at increased risk of CRC. Longitudinal effects maintained that Hispanic-related risk worsened with time in CRC mortality. Associations between SES and CRC mortality remained and appeared to grow with time as shown by moderation associations between SES and time.

Accounting for moderation between SES and preventive factors (Model 3) revealed that associations between SES and CRC mortality increased with increasing rates of colonoscopy uptake. Indeed, when adjusting for colonoscopy usage, counties with higher SES and with higher blood stool testing alone showed elevated risk of CRC mortality suggesting that SES helps to moderate the base effect of medical risk factors in these data.

## 5 Discussion

Fundamental cause theory suggests that social inequalities arise, in part, because social actors utilize resources to improve survival for their own families and communities. Prior work has found that social inequalities in CRC mortality exist and are growing steadily (Saldana-Ruiz et al., 2013). This study examined whether socioeconomic and racial/ethnic inequalities would grow when one particular type of screening was more effective than alternatives, and thus were interested in whether individuals are broadly able to utilize different mechanisms and we also examined whether SES and/or race/ethnicity influences the effectiveness of those mechanisms when multiple medical screening methods are available. We found that in an era when uptake of medical screening was increasing and prevalence of behavioral risk factors was declining, the benefits of those changes were concentrated among individuals living in higher SES areas.

### 5.1 Implications

Current efforts to distribute technologies may be inefficient: many at-risk individuals may miss opportunities for prevention. Indeed, only approximately two-thirds of the sample had, at any point in their lives, a colonoscopy, and many had poor health behaviors including physical inactivity. Efforts to control CRC should continue to improve screening rates in populations at risk for inadequate screening, and further in populations such as Native Americans who had similar overall screening rates but much higher risk of CRC mortality. Results further noted that colonoscopy, and not blood stool testing, was associated with drastically improved CRC mortality rates. However, when accounting for blood stool testing usage, these rates were preferentially lowered in high-SES areas suggesting that higher SES actors are more effective at accessing higher quality screening techniques when only one screening technology is used. In contrast, SES-related inequalities were reduced in areas that relied on blood stool testing, in part because it is a less effective tool for reducing the risk of CRC mortality.

Fundamental cause theory has provided a scaffold on which to build our understanding of how social conditions influence cause-specific mortality in general, and CRC in particular. Addressing causality, one randomized control trial found that social inequalities emerged in the treatment group after respondents were provided with information about breastfeeding (Yang et al., 2014). Resources, notes the theory, can be useful in helping individuals and communities curtail the risk of risks and are thus increasingly fungible in health domains. However, increasingly the literature notes that social inequalities may, at once, be increasing and decreasing (Krieger et al., 2012), in part because social inequalities are situated in a history where mechanisms are fluidly changing their effectiveness in both reducing mortality and in increasing social inequalities (Clouston et al., 2016; Miech et al., 2011). This study documented one such mechanism that was becoming more effective in predicting socioeconomic inequalities over time.

While the above extension is important, this paper has at least two novel substantive results. Specifically, this study responds to an expressed need to examine mechanisms linking domains of social inequality with health outcomes (Galea and Link, 2013). Indeed, it is one of the only studies to systematically examine a range of mechanisms linking socioeconomic and racial/ethnic inequalities with any reason for cause-specific mortality, though studies examining behavioral risk factors as a mechanism linking socioeconomic status to health and mortality are becoming increasingly common (Nandi et al., 2014; Stringhini et al., 2017). This work therefore builds on existing work that describes and clarifies the extent to which inequalities are evident in health (Link et al., 2008; Link and Phelan, 2010; Mackenbach et al. 2004, 2008, 2015; Phelan et al., 2004; Phelan and Link, 2005), but also extends it by examining how such inequalities arise for a particular cause of death and by comparing different mechanisms in a single moderated environment. This effort helps to clarify the mechanisms that may be particularly important to CRC mortality, but also highlights that both behaviors and medical preventions can work concurrently to create social inequalities in health.

Colorectal cancer is an optimal cause of death with which to understand causal mechanisms linking SES and race/ethnicity to mortality for at least three reasons. First, CRC is a common cause of death that accounts for as much as 50,000 deaths each year and is attributable to 8% of all American deaths (Centers for Disease Control and Prevention, 2017). Since disease prevention efforts, when effective, have been long considered a critical entry-point for inequalities to occur (Clouston et al., 2016), CRC is a fascinating case because despite being a common killer, it is highly preventable, with screening efforts alone reducing risk by more than 50% (Atkin et al., 2010). At the same time, CRC offers a unique vision on the creation and maintenance of inequalities, as well as on different types of ongoing mechanisms because it is not perfectly controlled by a single medication. This provides a unique opportunity to explore multiple overlapping mechanisms, including behavioral, that have been employed effectively at different stages in the disease process to influence risk.

### 5.2 Limitations

While we made a number of inroads into clarifying mechanisms linking SES and race/ethnicity with CRC mortality, this effort is limited in a number of ways. First, we could not examine whether individual risk for CRC changed due to processes external to those observed here including, for example, changes in red meat intake or differences in probiotic intake (Asha and Gayathri, 2012). Additionally, while data from the BRFSS and CMF starting in 1999 include information about Race and Hispanic ethnicity, CMF data prior to 1999 do not, resulting in a limited examination of differences prior to that time. Data were also measured by two different agencies using different metrics and incorporated different levels of analysis resulting in a limited ability to examine heterogeneity at the county level when examining preventive and behavioral risk factors. Behavioral risk factors were measured using self-report and are therefore subject to both recall bias and to differences in the acceptability of reporting known poorer health behaviors. Generalizability of these analyses may be limited both in their geographic focus on the United States, and in their application to small subregions that were excluded from analysis. Data were measured at the ecological level and thus it was impossible to state explicitly that those engaging in a specific preventive or risk factor were also those who benefited, reducing our ability to ensure causal associations between mechanisms. There is a potential for over-adjustment when controlling for multiple constructs at the same time. We attempted to avoid over-adjustment by separating SES models into individual and geographic analyses. However, while individual SES are associated with county-level SES there is no guarantee that individuals within geographic analyses that geographic markers of SES are strongly associated with individual-level measures of education and/or income since these measures incorporate, to a great extent, specific information about an individual's context. Thus, while there is some potential for over-adjustment there is also a need to understand the extent to which individual choices versus social context may improve our understanding. Finally, there was no consideration herein for how CRC histology or location may differ by socioeconomic factors. To compensate for these limitations, we merged data at the state-level, where both data sources are representative and comparable, and incorporated analyses of individual-level predictors of behavioral risk factors and uptake of prevention in order to clarify the mechanisms through which SES and race may operate. We further referenced a host of studies that clarify the role of each risk factor since it was not our intention to establish causality of any particular mechanism, but instead to note that SES and racial inequalities are worse in places where previously established interventions are readily available. Additionally, since CRC is rare and studies of socioeconomic inequalities in this area are likely to be underpowered by a lack of observation, we note that the novel data resources used were uniquely powered to examine these questions. Future work should, nevertheless, examine other potential databases that may allow further individual-level analyses of pathways linking SES and race/ethnicity with CRC mortality.

### 5.3 Policy implications

Despite enormous changes in ability to prevent CRC, recent reports suggest that many states have not experienced reduced CRC risk (Clouston et al., 2017). One reason for such limitations may be that they do not consider the role of financial risk. For example, while colonoscopy is generally covered by health insurers as a preventive procedure, when they detect polyps or malignancies they may be reclassified into diagnostic tests, requiring some patients cover up to 20% of the billed cost (estimated at $3,855–5,055 in 2013) (Centers for Medicare and Medicaid Services, 2013). The risk that individuals might need to pay $1,000–1,500 given a high detection rate may reduce the acceptability of the procedure in low-income settings (Corley et al., 2014). Additional costs may accrue from increased transportation costs, opportunities costs, and from other social and economic costs.

Everyone deserves the chance to be healthy (Braveman, 2006), and understanding how social inequalities in CRC arise is paramount to implementing effective interventions. Physicians and policymakers are increasingly charged with intervening on fundamental causes of health (Reich et al., 2016). Studies have reported successfully reducing inequalities via utilization of clinical outreach programs that target barriers to access (Lane et al., 2013) or uniform treatment regimens (Hernandez, 2013). However, while it is clear that inequalities can be addressed in such a way, systematic efforts to reduce inequalities cannot rely solely on demand-side factors to distribute technologies. For example, in a national program to provide free cancer screening to 2.6 million residents of the United Kingdom (von Wagner et al., 2011), CRC screening was freely provided but nearly half (46%) of potential participants did not use the screening program, and uptake was much larger in higher versus lower SES groups. Relying on single-pronged solutions is insufficient to this task: when distributional efforts are left to regional health authorities to manage, and for individuals to learn about, social inequalities appear likely to develop as areas with more resources increase capacity while others are left behind.

## Declarations

### Author contribution statement

S. Clouston: Conceived and designed the experiments; Performed the experiments; Analyzed and interpreted the data; Contributed reagents, materials, analysis tools or data; Wrote the paper.

J. Acker and M. Rubin: Conceived and designed the experiments; Analyzed and interpreted the data; Contributed reagents, materials, analysis tools or data; Wrote the paper.

B. Link: Conceived and designed the experiments; Wrote the paper.

D. Chae: Analyzed and interpreted the data; Wrote the paper.

### Funding statement

This research did not receive any specific grant from funding agencies in the public, commercial, or not-for-profit sectors.

### Competing interest statement

The authors declare no conflict of interest.

### Additional information

No additional information is available for this paper.

## Appendix A Supplementary data

Table S1_V2 Table S1_V2
