# Supplementary material for: Fundamental social causes of inequalities in colorectal cancer mortality: A study of behavioral and medical mechanisms
Source: Heliyon. 2020 Mar 11;6(3):e03484. doi: 10.1016/j.heliyon.2020.e03484 (PMC7068626; doi:10.1016/j.heliyon.2020.e03484)
Supplement: Table S1_V2 [file mmc1.docx]

**Table S1.** Colorectal cancer deaths, midyear population estimates, and crude mortality rates for U.S. residents aged 25 and older, Compressed Mortality File 1988-2012

|  | White | | |  | Black | | |  | American Indian/Alaskan Native | | |  | Asian and Pacific Islander | | |  | Hispanic | | |
| --- | --- | --- | --- | --- | --- | --- | --- | --- | --- | --- | --- | --- | --- | --- | --- | --- | --- | --- | --- |
|  | Colorectal Cancer Deaths (thousands) | Population (millions) | Crude Mortality Rate (/100,000) |  | Colorectal Cancer Deaths (thousands) | Population (millions) | Crude Mortality Rate (/100,000) |  | Colorectal Cancer Deaths (thousands) | Population (millions) | Crude Mortality Rate (/100,000) |  | Colorectal Cancer Deaths (thousands) | Population (millions) | Crude Mortality Rate (/100,000) |  | Colorectal Cancer Deaths (thousands) | Population (millions) | Crude Mortality Rate (/100,000) |
| 1999 | 44.628 | 159.631 | 27.96 |  | 6.655 | 25.369 | 26.23 |  | 0.168 | 1.631 | 10.30 |  | 0.820 | 8.634 | 9.50 |  | 4.884 | 23.820 | 20.50 |
| 2000 | 44.670 | 160.014 | 27.92 |  | 6.789 | 25.671 | 26.45 |  | 0.191 | 1.686 | 11.33 |  | 0.917 | 8.972 | 10.22 |  | 4.910 | 24.771 | 19.82 |
| 2001 | 43.799 | 160.949 | 27.21 |  | 6.770 | 26.200 | 25.84 |  | 0.189 | 1.718 | 11.00 |  | 1.006 | 9.507 | 10.58 |  | 5.140 | 26.145 | 19.66 |
| 2002 | 43.375 | 161.569 | 26.85 |  | 6.780 | 26.608 | 25.48 |  | 0.241 | 1.745 | 13.81 |  | 1.019 | 9.918 | 10.27 |  | 5.338 | 27.221 | 19.61 |
| 2003 | 42.785 | 162.124 | 26.39 |  | 6.859 | 27.004 | 25.40 |  | 0.199 | 1.772 | 11.23 |  | 1.078 | 10.312 | 10.45 |  | 5.037 | 28.267 | 17.82 |
| 2004 | 40.479 | 162.839 | 24.86 |  | 6.578 | 27.477 | 23.94 |  | 0.220 | 1.800 | 12.22 |  | 1.076 | 10.706 | 10.05 |  | 5.425 | 29.332 | 18.50 |
| 2005 | 39.980 | 163.594 | 24.44 |  | 6.805 | 27.977 | 24.32 |  | 0.231 | 1.830 | 12.62 |  | 1.102 | 11.116 | 9.91 |  | 5.132 | 30.480 | 16.84 |
| 2006 | 40.085 | 164.336 | 24.39 |  | 6.784 | 28.480 | 23.82 |  | 0.220 | 1.858 | 11.84 |  | 1.152 | 11.529 | 9.99 |  | 5.321 | 31.661 | 16.81 |
| 2007 | 40.028 | 164.955 | 24.27 |  | 6.800 | 28.957 | 23.48 |  | 0.245 | 1.883 | 13.01 |  | 1.204 | 11.928 | 10.09 |  | 5.305 | 32.826 | 16.16 |
| 2008 | 39.163 | 165.523 | 23.66 |  | 6.854 | 29.427 | 23.29 |  | 0.301 | 1.906 | 15.79 |  | 1.329 | 12.323 | 10.78 |  | 5.678 | 34.006 | 16.70 |
| 2009 | 38.286 | 166.015 | 23.06 |  | 6.722 | 29.871 | 22.50 |  | 0.292 | 1.926 | 15.15 |  | 1.249 | 12.707 | 9.83 |  | 5.853 | 35.165 | 16.64 |
| 2010 | 38.834 | 166.344 | 23.35 |  | 6.945 | 30.194 | 23.00 |  | 0.274 | 1.940 | 14.12 |  | 1.455 | 12.993 | 11.20 |  | 5.115 | 36.047 | 14.19 |
| 2011 | 38.266 | 167.030 | 22.91 |  | 6.924 | 30.721 | 22.54 |  | 0.314 | 1.971 | 15.93 |  | 1.443 | 13.501 | 10.69 |  | 5.352 | 37.191 | 14.39 |
| 2012 | 37.885 | 167.556 | 22.61 |  | 6.787 | 31.147 | 21.79 |  | 0.289 | 1.998 | 14.46 |  | 1.547 | 13.926 | 11.11 |  | 5.524 | 38.118 | 14.49 |
|  |  |  |  |  |  |  |  |  |  |  |  |  |  |  |  |  |  |  |  |
| Total | 572.263 | 2292.479 | 24.96 |  | 95.052 | 395.104 | 24.06 |  | 3.374 | 25.666 | 13.15 |  | 16.397 | 158.071 | 10.37 |  | 74.014 | 435.051 | 17.01 |
